# Water, sanitation and hygiene: measuring gender equality and empowerment

**DOI:** 10.2471/BLT.18.223305

**Published:** 2019-05-14

**Authors:** Georgia L Kayser, Namratha Rao, Rupa Jose, Anita Raj

**Affiliations:** a Division of Global Health, Department of Family Medicine and Public Health, School of Medicine, University of California, San Diego, 9500 Gilman Drive, La Jolla, CA 92093, United States of America (USA).; b Center on Gender Equity and Health, Department of Medicine, University of California, San Diego, USA.

The right to water and sanitation is recognized as fundamental to attaining all other human rights. Globally, however, 2.1 billion people do not have access to safe drinking water at home, 2.3 billion do not have basic sanitation and 1 billion practice open defecation.^1^ Women and girls are disproportionately affected by the lack of access to basic water, sanitation and hygiene facilities, due to their needs during periods of increased vulnerability to infection around menstruation and reproduction.^2^^–^^4^ Women and girls also have a larger role relative to men in water, sanitation and hygiene activities, including in agriculture and domestic labour.^4^ This situation has implications for gender equality and empowerment. The sustainable development goals (SDGs) make an important initial step in connecting water, sanitation and hygiene (SDG 6) and gender equality and empowerment (SDG 5) through target 6.2, which emphasizes access to equitable sanitation and hygiene, and the needs of women and girls. However, indicators to measure the specific needs of women and girls are still emerging.

Inequalities in access to water, sanitation and hygiene services have been measured between rural and urban areas and across country wealth quintiles, as well as by sex. However, measurement of the burden placed on women and girls, the opportunity costs of these burdens, and female empowerment related to water, sanitation and hygiene decision-making and autonomy are limited.^1^^,^^5^ Based on a process of expert input and literature review, here we offer a compilation of current water, sanitation and hygiene indicators that measure gender equality and empowerment in four interrelated priority areas. Within each priority area, we describe and critique the status of these measures and identify where further research is needed to better measure gendered aspects of water, sanitation and hygiene at programme, national and global levels.

The first priority area is that of women’s water-fetching responsibility and time-use burden, and the implications for health and economic well-being. Women and girls are responsible for fetching water in four out of five households where a drinking water source is off premises.^1^^,^^4^^,^^6^ This practice has implications for women’s health in the form of spinal injury, neck pain, spontaneous abortion from heavy and awkward workloads, and caloric expenditure.^1^^,^^6^ However, there is limited research to quantify the full burden associated with water carriage. When girls carry water over long distances, the time available to them to pursue education is reduced.^1^ Water-fetching responsibilities also add to the burden of unpaid domestic work, decrease time towards other income-generating activities and affect the time for leisure and nonessential activities. Further research on these issues is needed.^6^

Currently, as part of SDG monitoring, indicators are collected on access to water on premises (SDG 6) and proportion of time spent on unpaid domestic and care work by sex and age (SDG 5). Time for fetching water is included in the SDGs as a basic water service within a 30-minute round-trip; yet, these data are rarely disaggregated by gender at the global level.^1^ Round-trip time to the water source, primary person responsible for water collection and the number of trips made by this person are collected by national-level Multiple Indicator Cluster Surveys in 100 countries, and can be disaggregated by gender and age.^5^ While much more could be done with existing data, measurement of time-use related to water fetching and whether this task reduces opportunities for attending school, income generation, child care, and safety risks is currently incomplete.

The second area is sanitation access and its relationship with gender-based violence and psychosocial stress. The growing evidence on sanitation-related gender-based violence highlights a range of vulnerabilities faced by women and girls who are forced to openly defecate or walk to shared sanitation facilities. This evidence describes the fear of sexual violence that can restrict freedom of movement and affect equal opportunities. 

Reports from Ethiopia,^7^ India,^3^^,^^8^ and refugee or internally-displaced persons camps in Guinea, Haiti, Kenya, Liberia, the Philippines, Sierra Leone and Somalia^9^ document physical and sexual harassment and assault by non-partners, fear of sexual violence and harassment and stress faced by women and girls while they access shared sanitation facilities or openly defecate.

More research on the prevalence of sanitation-related gender-based violence and the relationship between water and sanitation access, gender-based violence and health is needed. Emerging research on water, sanitation and hygiene insecurity and mental health in women in Ethiopia found that water insecurity was predictive of psychological distress, as measured by the World Health Organization (WHO) self-reporting questionnaire.^7^ Research in rural India on a sanitation insecurity measure that evaluates sanitation interventions for physical and social context found that unmarried women and women without a functioning latrine had a higher sanitation insecurity score, which in turn was associated with distress and poorer mental health.^8^

Further research is needed on sexual harassment to understand where women and girls feel unsafe when they use such facilities; which water, sanitation and hygiene designs could increase safety; the relationship between distance to these facilities and feelings of insecurity, prevalence of various types of harassment and violence, nature of perpetrator and mental and physical health outcomes. Furthermore, women and girls’ participation in decision-making on water, sanitation and hygiene design, maintenance, service availability and pricing should be better monitored, and implications for safety and security studied.

The third area is women’s water, sanitation and hygiene needs during menstruation, pregnancy and caregiving, and effects on health, education and psychosocial stress. Women have an increased need for water for hydration, sanitation and hygiene during menstruation, pregnancy, the postnatal period and while caring for sick family members or young children.^4^^,^^9^ When these basic needs are not met, women and girls are unable to participate equally in society. 

The standard water indicators are whether households had sufficient water to meet needs during the past month and how many months out of the year water is available at the main water source. Both of these indicators are collected as part of the Multiple Indicator Cluster Survey and assessed by the respondent.^5^ Quantity (litres per person per day) is not currently measured in monitoring at any level, and normative guidelines for water quantity from WHO are related to emergencies.^5^

Research in India points to a strong association between open defecation and adverse pregnancy outcomes, even after controlling for sociodemographic and clinical factors. The findings suggest that private household water and sanitation that decreases open defecation and time travelled to the water source could help to reduce preterm birth and low birth rate.^2^ Further research could expand on water, sanitation and hygiene requirements to satisfy domestic, reproductive and sexual health needs. Research could disaggregate the data by gender, life stage (pregnant and nursing mothers), physical exercise or work exertion and climatic zone to better understand requirements across the life course.

The lack of basic water, sanitation and hygiene services in households and extra-household settings, such as schools and health-care facilities, has implications for girls’ menstrual hygiene management, safety and emotional and physical well-being. The presence of these services in health-care facilities is critical to reducing the risk of infections among patients and providers, especially during childbirth. 

In many low- and middle-income countries, water, sanitation and hygiene in health-care facilities are far from adequate.^1^ In schools, the lack of basic infrastructure, privacy, spaces, materials and guidance to manage menstruation has been associated with harassment, sexual exploitation, psychosocial impacts, decreased school attendance rates and drop-out for girls.^9^ However, further quantitative research is needed across settings. 

Within the framework of the SDGs, basic access to water, sanitation and hygiene in schools and health-care facilities will be measured across countries, including the proportion of schools with improved sanitation that is single-sex and usable. More cross-country data is needed on the impact of girl student-to-latrine ratios, presence of waste disposal options and/or washing rooms close to sanitation facilities, access to menstrual management materials, and girls’ attitudes, self-efficacy and agency around water, sanitation and hygiene and menstrual hygiene management access in household and external settings.

The fourth priority area is that of women’s participation in water, sanitation and hygiene decision-making and governance, leading to their social and political empowerment. The central role of women and girls in the procurement and management of water, sanitation and hygiene at the household level is recognized. However, measures of women’s participation in the governance and household decision-making control over such resources is scarce, although such participation may result in more gendered considerations in addressing some of the mentioned issues related to access and safety.^4^^,^^10^ One multicountry study found women’s participation in key water committee positions of leadership to be a significant predictor of functional and sustainable water systems.^10^ There is evidence that empowering women to make sanitation decisions can enhance performance outcomes for the household and community.^11^ Research in Kenya revealed that women’s decision-making power for major household purchases was positively associated with households with latrine ownership.^11^ Cross-country measurement is needed on the proportion of local water, sanitation and hygiene governance bodies with female representatives.^5^^,^^7^^,^^12^

Women are largely responsible for household water, sanitation and hygiene management; they bear a disproportionate burden when these basic services are lacking, and face health, security and psychological vulnerabilities due to inadequate access and decision-making control. We emphasize the need for measurement at the intersection of gender equality and water, sanitation and hygiene to guide SDG monitoring and achievement.
